# Evaluation of Antioxidant and Anti-Inflammatory Effects of a Nanoformulation Derived from Annurca Apple Callus Extract in an In Vitro Model of Iron Overload-Induced Inflammation

**DOI:** 10.3390/antiox14060631

**Published:** 2025-05-24

**Authors:** Federica Gubitosa, Laura Taramova, Stefanie Ho Yi Chan, Joan Liu, Daniele Fraternale, Vinood B. Patel, Satyanarayana Somavarapu, Lucia Potenza, Mohammed Gulrez Zariwala

**Affiliations:** 1 Department of Biomolecular Sciences, University of Urbino Carlo Bo, 61029 Urbino, Italy; f.gubitosa@campus.uniurb.it (F.G.); daniele.fraternale@uniurb.it (D.F.); lucia.potenza@uniurb.it (L.P.); 2 Centre for Nutraceuticals, School of Life Sciences, University of Westminster, 115 New Cavendish Street, London W1W 6UW, UK; l.taramova1@westminster.ac.uk (L.T.); s.chan4@westminster.ac.uk (S.H.Y.C.); v.b.patel@westminster.ac.uk (V.B.P.); 3 Department of Pharmaceutics, UCL School of Pharmacy, 29-39 Brunswick Square, London WC1N 1AX, UK; s.somavarapu@ucl.ac.uk

**Keywords:** ferroptosis, nanoformulation, skin protection, antioxidant, natural compounds

## Abstract

Ferroptosis, a regulated form of cell death driven by iron accumulation and lipid peroxidation, contributes to oxidative stress-related skin damage. This study evaluates the antioxidant and anti-inflammatory effects of a nanoformulation derived from an Annurca apple callus extract in an in vitro model of ferroptosis using human keratinocytes (HaCaT cells). A hydroalcoholic extract from light Annurca apple callus (LCE) was nanoformulated with Pluronic^®^ F127 and Soluplus^®^ to enhance stability and bioavailability. The resulting nanoformulation (NF-LCE) exhibited optimal particle size (103.17 ± 0.87 nm), polydispersity index (0.21 ± 0.00), and zeta potential (−1.88 ± 0.64 mV). Iron overload (100 µM) was employed to induce oxidative stress and inflammation in HaCaT cells, resulting in elevated levels of inflammatory markers (COX2, IL-6, TNF-α) and a diminished antioxidant response, as indicated by decreased expression of GPX4 and Nrf2. NF-LCE treatment restored GPX4 and Nrf2 levels (~0.8-fold increase, *p* < 0.05) while significantly reducing COX2 (36.6%, *p* < 0.01), IL-6 (79.6%, *p* < 0.0001), and TNF-α (30.9%, *p* < 0.1). These results suggest NF-LCE as a promising therapeutic strategy for mitigating ferroptosis-induced skin damage, warranting further investigation in advanced skin models and clinical applications.

## 1. Introduction

Skin is the body’s largest organ, protecting the body from environmental and xenobiotic agents. At the same time, it insulates the body and regulates its temperature, avoiding an uncontrolled loss of water and solutes [1,2]. This makes the skin the most exposed organ to environmental factors such as pollution, temperature, and sunlight radiation, which can contribute to the skin’s intrinsic and extrinsic ageing [3]. Although many environmental and genetic factors contribute to the development of various skin diseases, the most important factor is chronic skin exposure to solar ultraviolet (UV) radiation. UV light-induced reactive oxygen species (ROS) generation is a significant mechanism that may negatively impact health [4].

Iron is vital for numerous biological processes. However, high levels of unbound iron can be detrimental as it increases the production of ROS via Haber–Weiss and Fenton reactions [5]. Intracellular iron levels are thus tightly regulated to prevent the generation of free radicals, which can compromise cell integrity [5].

Ferroptosis is a recently identified form of programmed cell death involving iron accumulation and consequent lipid peroxidation [6]. It is characterised by distinct morphological, biochemical, and genetic traits that differentiate it from other established forms of regulated cell death [7]. Ferroptosis plays a crucial role in a variety of conditions, including neurodegenerative diseases, tumours, myocardial injury, fibrosis, and potentially skin diseases [8,9]. Keratinocytes, which comprise over 90% of epidermal cells, absorb around 95% of UV light that reaches the skin [10]. When skin cells are exposed to ultraviolet A (UVA) radiation (320–400 nm), there is an immediate increase in the intracellular labile iron (LI) pool, heightening cellular susceptibility to oxidative damage and potential cell death via ferropototic mechanisms. The ROS generated by UVA exposure directly harms skin components, leading to an increased cytosolic LI pool that further exacerbates oxidative damage [5,11]. While various protective antioxidant enzymes such as superoxide dismutase (SOD), catalase (CAT), and glutathione peroxidase (GPx) are present as part of cellular antioxidant defence systems, recent trends have highlighted the benefits of using exogenous antioxidants. Studies indicate that plants are a rich source of natural protective molecules with anti-ageing properties that have been shown to stimulate immune responses, mitigate inflammation, bolster antioxidant defences, detoxify cells and tissues, and influence gene expression [12,13,14]. As a result, they restore redox homeostasis and protect the skin from intrinsic chronological ageing and extrinsic photoaging [12].

Triterpenes are among the most abundant natural products, with approximately 30,000 known structures. Common in medicinal plants and foods like fruits, vegetable oils, and cereals, these compounds are noted for their anticancer, anti-inflammatory, antioxidant, antiviral, and antidiabetic effects [15,16]. Pentacyclic triterpenes have garnered significant interest for their therapeutic potential and are widely marketed as dietary supplements and treatments. Triterpenic compounds are well-regarded for their effectiveness in topical formulations addressing various skin conditions, from ageing to malignant melanoma [17,18,19,20,21]. However, their high hydrophobicity limits their effectiveness as pharmacological agents by restricting skin penetration [22]. Consequently, multiple strategies have been developed to address this limitation [17,23,24,25,26]. Recent advancements in pharmacology and nanotechnology have introduced nanocarriers as innovative drug delivery systems. Nanoparticles’ size and functional versatility enhance pharmacological benefits by improving stability and cellular delivery. In dermatology, nanocarrier-based systems notably improve skin penetration and allow for precise cellular targeting [27,28].

In a previous study, Gubitosa et al. [29] developed two callus cultures from the ripe pulp of the Annurca Apple, grown under distinct light conditions: one in complete darkness and the other under an 18 h photoperiod. The resulting hydroalcoholic extracts were chemically characterised, and the potential biological activity was assessed. The findings indicated that these extracts are rich in triterpenic compounds, with tormentic acid (TA) identified as the most abundant compound in the chemical composition and demonstrating significant anti-inflammatory properties [29].

This study sought to develop a nanoformulation incorporating either the hydroalcoholic extract of the 18 h photoperiod developed LCE or pure standard TA as a reference and assess the anti-inflammatory potential of both the nanoformulated compounds and their unformulated counterparts in a human keratinocyte preliminary iron-overload-induced inflammation model.

## 2. Materials and Methods

### 2.1. Reagents and Materials

Ferrous sulfate heptahydrate (FeSO_4_·7H_2_O) was used as the pro-oxidant agent in all iron-related experiments. All cell culture reagents were of cell culture grade and, unless otherwise specified, were purchased from Sigma-Aldrich (Dorset, UK). The protease inhibitor cocktail (PIC), dimethyl sulfoxide (DMSO), 3-(4,5-dimethylthiazol-2-yl)-2,5-diphenyltetrazolium bromide (MTT), and other chemicals were also supplied by Sigma-Aldrich. Culture media, Minimum Essential Medium (MEM) and Dulbecco’s modified Eagle’s medium (DMEM)-GlutaMax, foetal bovine serum (FBS), and 100× antibiotic-antimycotic solution were obtained from Fisher Scientific (Loughborough, UK). Cell culture plates and flasks (6- and 96-well) were provided by Nunc (Roskilde, Denmark).

### 2.2. Preparation of LCE

The callus derived from the Annurca apple (Annurca Apple Light Callus) was obtained from the Botanical Garden of the University of Urbino Carlo Bo, following the protocol described by Verardo et al. [30]. The extract (LCE) was prepared according to the method of Gubitosa et al., 2024 [29], with slight modifications. A total of 200 mg of freeze-dried sample was dissolved in 11.5 mL of 70% ethanol and homogenized using a Potter-type homogenizer (Steroglass, Perugia, Italy). The mixture was shaken overnight at 4 °C and then centrifuged at 13,000 rpm for 45 min at 4 °C. The supernatant was subjected to a second centrifugation at 4000 rpm for 30 min. The pellet was re-extracted with an additional 11.5 mL of 70% ethanol, shaken for 1 h, and centrifuged again at 13,000 rpm for 45 min. After a final centrifugation at 4000 rpm for 30 min, all supernatants were combined, filtered through a 0.45 µm filter, and concentrated using Rotavapor R-215 (Büchi Labortechnik AG, Flawil, Switzerland). The resulting material was dissolved in 20 mL of deionized water and lyophilized using a FreeZone Freeze Dryer (Labconco Corporation, Kansas City, MO, USA). The freeze-dried extract was stored at −20 °C until use.

### 2.3. Preparation of Nanoformulations

Nanoformulations were produced using a modified thin-film hydration technique. TA (1.15 mg, BLD Pharm, Shanghai, China), or an equivalent volume of LCE containing the same amount of TA, was dissolved in 20 mL of methanol together with Pluronic^®^ F127 (75%) (Sigma Aldrich, Welwyn Garden City, UK) and Soluplus^®^ (25%) (BASF, Stockport, UK). The content of TA in the LCE was quantified using HPLC-DAD prior to the formulation process, allowing precise control of its dosage. The solvent was removed using rotary evaporation (200 rpm, 60 °C, under vacuum) (Buchi Rotavapor^®^ R-100, Flawil, Switzerland), forming a thin film. This film was rehydrated with 10 mL of Milli-Q water at 60 °C. The dispersion was sonicated and filtered through a sterile 0.45 μm membrane to remove any unloaded active ingredients. Blank formulations were prepared following the same method but without the addition of TA or LCE.

### 2.4. Evaluation of the Physiochemical Properties of the Nanoformulations

The measurement of particle size and zeta potential was performed using dynamic light scattering (DLS). The size distribution and zeta potential of nanoparticles were determined with the Zetasizer Ultra (Malvern Instruments, Malvern, UK). This device provided the Z-average hydrodynamic diameter, polydispersity index (PDI), and zeta potential (§). Before measurement, samples were filtered through a 0.45 µm membrane. Subsequently, 1 mL of the sample was pipetted into the zeta potential folded capillary cell DTS1070 (Malvern Panalytical, Malvern, UK). The zeta potential was calculated from the electrophoretic mobility using the Helmholtz–Smoluchowski equation, processed by the Malvern data analysis software, version 5.3. Each sample underwent three measurements, and the mean values and standard deviations (SD) for particle size, PDI, and zeta potential were computed [31].

### 2.5. Morphology of Nanoparticles by Transmission Electron Microscopy (TEM)

The nanoparticles’ morphology was examined with TEM (Philips/FEI CM120 Bio Twin, FEI Company, Eindhoven, The Netherlands). Samples were placed on copper grids, and excess droplets were blotted away with filter paper. After four minutes, a drop of 1% phosphotungstic acid was added to the copper grid for negative staining. The grid was then air-dried and analysed using TEM.

### 2.6. Cell Culture

Immortalized human keratinocyte cells (HaCaT) were cultured in DMEM-GlutaMax^®^ supplemented with 10% FBS, 1% antibiotic/antimycotic, and 1% L-glutamine. Cells were maintained in a humidified atmosphere (95%) at 37 °C with 5% CO_2_, and media were refreshed every 2–3 days. Cells were seeded in 96-well or 6-well plates until approximately 80% confluence was reached.

### 2.7. Cell Viability Assay

An MTT assay was conducted to evaluate the potential cytotoxic effects of the test samples (TA, LCE, NF-TA, and NF-LCE) on HaCaT cells. Cells were seeded at a density of 30,000 cells/cm^2^ in 96-well microplates and cultured in complete DMEM—GlutaMax^®^ at 37 °C in a humidified atmosphere containing 5% CO_2_ until reaching confluency. Subsequently, the cells were treated with various concentrations of each sample, standardized to TA content, for 24 and 48 h. Untreated cells maintained in MEM served as the control. At the end of each incubation period, 20 µL of MTT solution (5 mg/mL in sterile DPBS) was added to each well [32]. After 4 h of incubation at 37 °C, the resulting formazan crystals were solubilized in 100 µL of DMSO following aspiration of the medium. To ensure complete dissolution, the plates were placed on a MaxQ 4000 orbital shaker (Thermo Fisher Scientific, Loughborough, UK) at 75 rpm for 15 min. Absorbance was then measured at 570 nm and compared to that of the untreated control cells (MEM).

### 2.8. Optimisation of an In Vitro Model for Iron-Overload-Induced Oxidative Stress

An in vitro iron-overload model was established by exposing HaCaT cells to increasing concentrations of iron (20 µM, 50 µM, 100 µM, and 200 µM), based on protocols adapted from previous studies [33,34,35,36]. Cells were seeded at a density of 10,000 cells/cm^2^ in 6-well plates and maintained at 37 °C with 5% CO_2_. Before iron treatment, the culture medium was replaced with serum-free MEM supplemented with 1% L-glutamine and 1% antibiotic-antimycotic solution, followed by a 24 h incubation. After this period, cells were washed with DPBS and treated with the indicated iron concentrations for 24 h. Subsequently, cells were washed again with DPBS and incubated in serum-free MEM for an additional 24 h. Finally, cells were lysed, and the resulting lysates were collected and stored at −20 °C. To rule out the possibility that increased iron concentrations might compromise cell viability, an MTT assay was performed at each treatment concentration.

### 2.9. Intracellular Total Iron Quantification

Intracellular total iron levels were quantified using a FerroZine™-based colorimetric assay, following the protocols described by Zariwala et al. [37] and Schiano et al. [38]. Briefly, 200 µL of 0.1 M HCl was added to 200 µL of cell lysate, while 200 µL of 50 mM NaOH was added to 200 µL of each iron standard solution (ranging from 0 to 120 µM). A blank was prepared using 400 µL of 50 mM NaOH. To each tube, 200 µL of a freshly prepared iron-releasing reagent—comprising equal volumes of 1.4 M HCl and 4.5% (*w*/*v*) KMnO_4_—was added. Samples were incubated in a dry bath at 60 °C under a fume hood for 2 h and then cooled to room temperature for 10 min. Subsequently, 60 µL of an iron detection reagent (containing 6.5 mM FerroZine™, 2.5 M ammonium acetate, and 1 M ascorbic acid in water) was added to each tube, followed by a 30 min incubation at room temperature. To assess colour development, 200 µL of each sample and standard was transferred in duplicate to a 96-well microplate, and absorbance was measured at 550 nm using a Fluostar Optima microplate reader (BMG Labtech, Offenburg, Germany). Intracellular iron concentrations were normalized to total protein content, determined using the bicinchoninic acid (BCA) assay.

### 2.10. Intracellular Ferritin Quantification

Ferritin content in HaCaT cells was measured using a commercial ELISA kit (Eagle Biosciences, Inc., Amherst, MA, USA), following the manufacturer’s instructions. Briefly, 20 µL of calibrators, controls, and cell lysate samples were dispensed in duplicate into a 96-well microplate. Subsequently, 200 µL of HRP-conjugate solution was added to each well, and the plate was incubated on a plate shaker at 200 rpm for 60 min at room temperature. After incubation, wells were washed five times with 300 µL of diluted wash buffer. Then, 150 µL of TMB substrate was added to each well, followed by a 30 min incubation on the plate shaker (200 rpm). The reaction was stopped by adding 50 µL of stop solution, and absorbance was measured at 450 nm within 20 min. Ferritin concentrations were normalized to total protein content, assessed via the bicinchoninic acid (BCA) assay.

### 2.11. Effects of Iron-Induced Inflammation in HaCaT Cells

HaCaT cells were seeded at a density of 10,000 cells/cm^2^ in 6-well plates and cultured until reaching confluence. Cells were then subjected to iron depletion by incubation in serum-free MEM for 24 h at 37 °C in a 5% CO_2_ atmosphere. Following this period, cells were washed with DPBS and treated for 3 h with 3.25 µM of LCE, TA, NF-LCE, or NF-TA, after which they were exposed to 100 µM iron for 24 h. At the end of the incubation, the medium was removed, and the cells were washed with DPBS. Cell lysates were collected using 350 µL of BB4 buffer (EasyPure^®^ RNA Kit, 50 rxns, TransGen Biotech, Beijing, China) and stored at −80 °C until further analysis.

### 2.12. Quantitative Real-Time PCR

Total RNA was extracted using the EasyPure^®^ RNA Kit (TransGen Biotech, Beijing, China). Subsequently, mRNA was reverse transcribed into cDNA and analysed by real-time PCR to assess the expression of GPX4, COX2, Nrf2, IL-6, and TNF-α, genes involved in both the inflammatory response and ferroptosis. Gene expression levels were normalized to the housekeeping gene 36B4. Quantitative PCR was performed in a 20 µL reaction volume containing 2 µL of cDNA, 10 µL of ChamQ Universal SYBR qPCR Master Mix, 0.4 µL each of forward and reverse primers (from a 10 µM stock), and 7.2 µL of nuclease-free water. Reactions were run on a T100 Thermal Cycler (Bio-Rad Laboratories, Inc., Hercules, CA, USA), and relative gene expression was calculated using the Pfaffl method [39]. Real-time PCR thermal cycles were carried out, as reported in Supplementary Table S1, and nucleotide sequences of the primers used are reported in Table 1.

### 2.13. Statistical Analysis

Statistical analyses were performed using GraphPad Software (GraphPad Prism version 8 for Windows, La Jolla, CA, USA). Values were expressed either as the mean ± standard error of the mean (SEM) or SD. The treated and control sample variables were compared using one-way ANOVA. Dunnett’s multiple comparisons test was employed to calculate the significant difference. The differences between samples were considered significant if *p* values were <0.05.

## 3. Results

### 3.1. Size, Charge, and Zeta Potential of Nanoformulations

The blank nanoformulation exhibited a mean particle size of 75.00 ± 0.47 nm, indicating a relatively small and uniform particle size distribution. NF-TA demonstrated a slightly increased mean particle size of 81.48 ± 0.39 nm, which may result from the interaction between TA and the nanocarrier matrix or polymers, leading to the formation of marginally larger particles. In contrast, NF-LCE showed a significantly larger mean particle size of 103.17 ± 0.87 nm. This substantial increase suggested that the bioactive compounds in apple extract contributed to the formation of larger particles, potentially due to particle aggregation or the entrapment of larger molecules inherent in the extract’s complex phytochemical composition. NF-LCE exhibited the highest PDI at 0.21 ± 0.00, indicating a broader size distribution. The heterogeneity in size distribution was likely caused by the complex nature of apple extract, which might lead to less uniform particle encapsulation or interactions within the nanocarrier matrix. However, all three formulations were considered monodisperse/uniform as the PDI obtained was below 0.25 (Table 2). All three nanoformulations were regarded as neutrally charged based on the zeta potential measurements (Table 2).

### 3.2. Morphological Characterization of Nanoparticles

**Figure 1:**
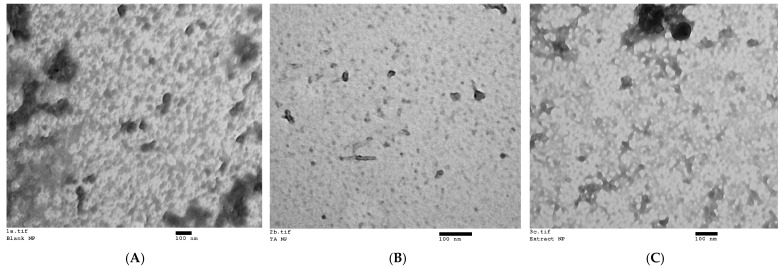
Transmission electron micrographs of nanoparticles of the three nanoformulations: (**A**) blank nanoformulation (magnification: 65 k); (**B**) NF-TA (magnification: 135 k); (**C**) NF-LCE (magnification: 93 k).

All formulations demonstrated a generally spherical morphology, with particles falling within the nanosize range in the TEM analysis. The particle sizes observed in the TEM images aligned with the mean particle sizes for each formulation, as determined by DLS (Figure 1).

### 3.3. Assessment of Cell Viability

**Figure 2:**
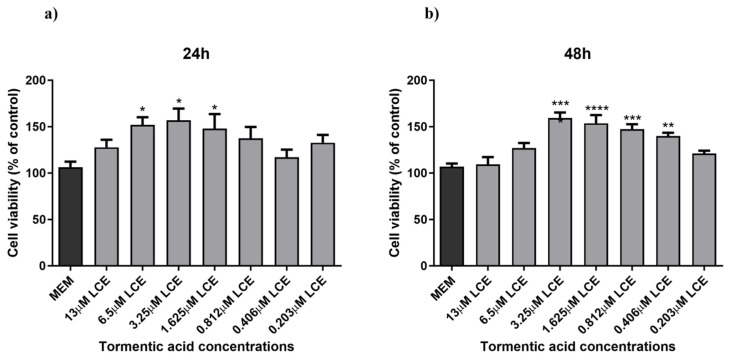
HaCaT cell viability. HaCaT cells were treated with different concentrations of LCE (13–0.203125 µM TA concentration) for 24 h (**a**) and 48 h (**b**), and cell viability was measured by MTT assay. MEM represents the control condition with cells incubated with treatment-free media. The results are expressed as% of CTRL ± SEM (*n* = 6; *p* ≤ 0.05). ANOVA followed by Dunnett’s multiple comparison test was performed (* *p* < 0.05, ** *p* < 0.01, *** *p* < 0.001, **** *p* < 0.0001).

A preliminary MTT assay was conducted to assess LCE’s toxicity and identify the optimal concentrations for nanoformulation. HaCaT cells were exposed to different LCE concentrations, expressed as TA concentrations:13 μM, 6.5 μM, 3.25 μM, 1.625 μM, 0.812 μM, 0.406 μM, and 0.203 μM for 24 and 48 h. As illustrated in Figure 2, all tested concentrations, except for 13 μM, were non-cytotoxic, with mean cell viability (±SEM) being 151.85 ± 18.53%, 156.89 ± 28.25%, 148.02 ± 34.70%, 137.40 ± 24.50%, 117.15 ± 18.15%, and 132.63 ± 14.63%, respectively. Interestingly, a significant increase (1.4-fold increase, *p*-value 0.0232) in cell viability was observed starting at a concentration of 6.5 μM.

**Figure 3:**
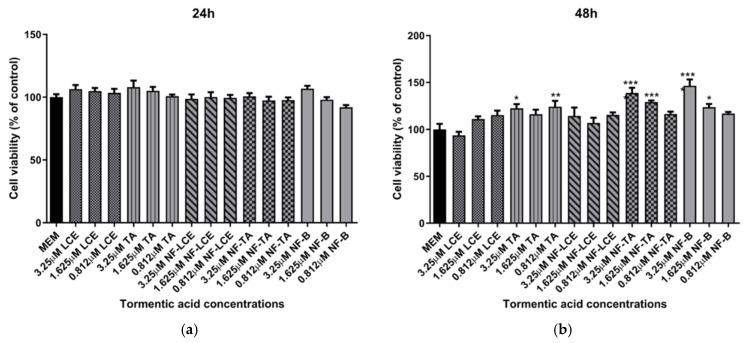
HaCaT cell viability after exposure to unformulated and nanoformulated compounds. HaCaT cells were treated with different concentrations of encapsulated and non-encapsulated LCE and TA for 24 h (**a**) and 48 h (**b**), and cell viability was measured by MTT assay. MEM represents the control condition with cells incubated with treatment-free media. The results are expressed as% of CTRL ± SEM (*n* = 6; *p* ≤ 0.05). ANOVA followed by Dunnett’s multiple comparison test was performed (* *p* < 0.05, ** *p* < 0.01, *** *p* < 0.001).

Based on these preliminary results, the concentrations of 3.25 μM, 1.625 μM, and 0.812 μM were selected for nanoformulation. Another series of experiments were performed comparing the effect of both formulated and unformulated LCE and TA, used as a reference compound, on cell viability. In this case, as shown in Figure 3, neither the pure compound nor the nanoformulations significantly affected the viability of HaCaT cells. This indicates that both treatments did not compromise cell viability, suggesting a favourable safety profile. Notably, slight differences in the cell viability results observed between Figure 2 and Figure 3, despite involving the same unformulated LCE concentrations, may be attributed to differences in experimental timing and context, as the two sets of experiments were conducted independently.

### 3.4. Assessment of the Iron-Overload Model

**Figure 4:**
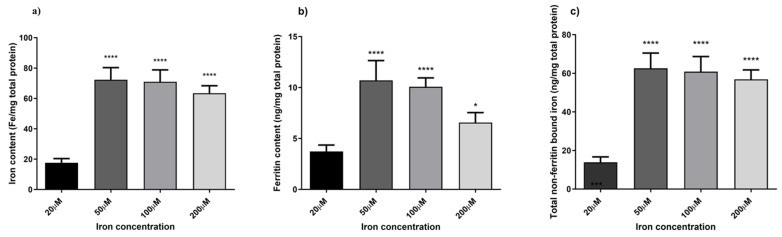
Quantification of non-bound and ferritin-bound iron levels. Intracellular iron accumulation was quantified using FerroZine^™^-based colourimetric assay (**a**) and Ferritin assay (**b**). Total non-ferritin-bound iron was obtained subtracting the total iron content obtained from the ferrozine assay from the corresponding mean ferritin concentrations (**c**). Data are expressed as mean ± SD *(n* = 6; *p* ≤ 0.05). ANOVA followed by Dunnett’s multiple comparison test was performed comparing each condition against the treatment condition with normal iron levels (20 µM) (* *p* < 0.05, **** *p* < 0.0001).

**Figure 5:**
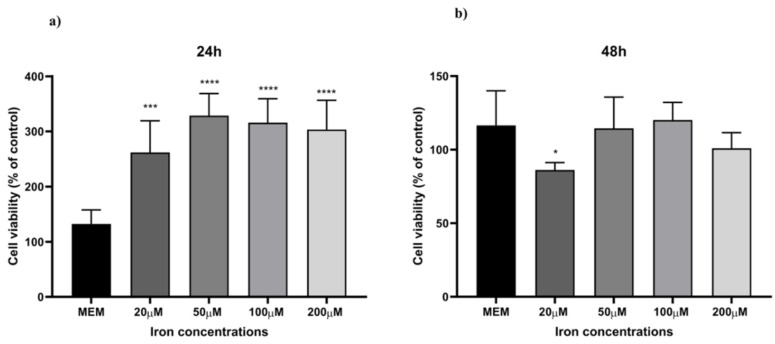
Cell viability. HaCaT cells were treated with different iron concentrations (20 µM, 50 µM, 100 µM, and 200 µM) for 24 h (**a**) and 48 h (**b**), and cell viability was measured by MTT assay. MEM represents the control condition with cells incubated with treatment-free media. Data are expressed as % of CTRL ± SD (*n* = 6; *p* ≤ 0.05). ANOVA followed by Dunnett’s multiple comparison test was performed (* *p* < 0.05, *** *p* < 0.001, **** *p* < 0.0001).

To optimise an in vitro iron-overload model, HaCaT cells were treated with different iron concentrations, ranging from normal to excessive prooxidant levels (20 µM, 50 µM, 100 µM, and 200 µM) for 24 h. Ferrozine and ferritin assays were used to evaluate iron accumulation within the cells by measuring non-bound and ferritin-bound iron levels. Figure 4 illustrates the correlation between iron concentration and iron accumulation in HaCaT cells. There is a progressive increase in iron accumulation with rising iron concentrations, reaching a maximum of 100 µM iron concentration (4.0-fold increase, *p* < 0.0001 detected with the Ferrozine assay and 2.7-fold increase, *p* < 0.0001 with the ferritin assay), after which iron accumulation decreases. Moreover, Figure 4c showed a trend between an increase in the LI pool and iron concentrations with total non-ferritin-bound iron in the range of 9.5 ng/mg to 54.8 ng/mg total protein from 20 µM to 200 µM, reaching a plateau at the 100 µM concentration (4.4-fold increase, *p* < 0.0001). These results are consistent with measured iron level results presented in studies carried out in other cellular models subjected to different oxidative stress stimuli [35,40,41,42]. Since the aim was to induce iron-related inflammation in HaCaT cells without affecting cell viability, a cell viability assay was conducted to assess the reliability of the cellular inflammatory response following the treatment with iron concentrations. As observed in Figure 5, cell viability was not significantly affected over the specified period.

### 3.5. Modulation of Gene Expression Following NF Treatment

**Figure 6:**
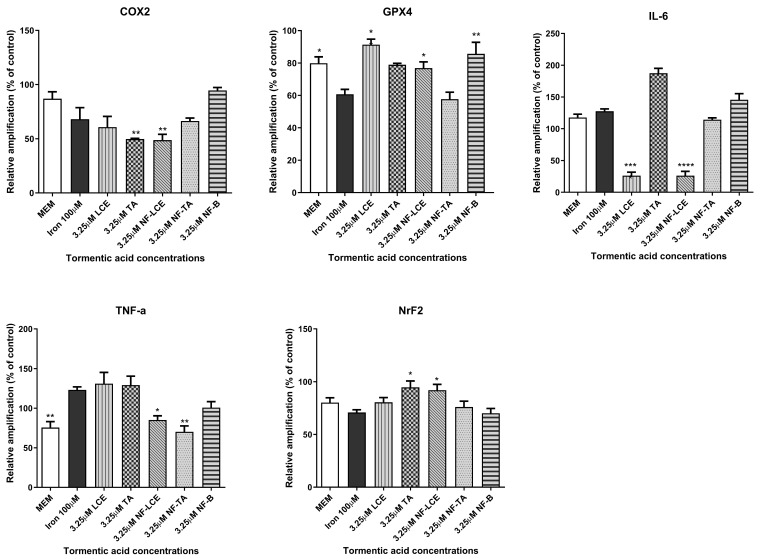
Anti-inflammatory activity of formulated and non-formulated LCE and TA on iron-treated HaCaT cells. HaCaT cells were pre-treated for 3 h with formulated and non-formulated LCE and TA (3.25 µM) followed by 24 h incubation with 100 µM Iron. MEM represents the control condition with cells incubated with treatment-free media. Data are expressed as% of CTRL ± SEM (*n* = 3; *p* ≤ 0.05). ANOVA followed by Dunnett’s multiple comparison test was performed against the prooxidant condition alone (100 µM Iron) (* *p* < 0.1, ** *p* < 0.01, *** *p* < 0.001, **** *p* < 0.0001).

After optimisation of the HaCaT ferroptosis model, the effect of iron on inflammation was assessed in HaCaT cells. The ability of LCE, TA, NF-LCE, and NF-TA to attenuate or reduce the inflammation by investigating the expression of genes. These included GPX4, which plays a crucial role in protecting cells from oxidative damage [43,44]; COX2, an enzyme involved in the inflammatory process [45]; and NrF2, a transcription factor that regulates the expression of antioxidant proteins [46]. Additionally, the expression of pro-inflammatory cytokines, specifically IL-6 and TNF-α, was evaluated [47]. Additionally, GPX4, Nrf2, and COX2 play specific roles in the induction of ferroptosis-related inflammation as well as the modulation of iron levels [48,49,50]. As illustrated in Figure 6, 100 µM iron exposure enhances the expression of COX2, IL-6, and TNF-α, whilst NF-LCE suppressed their expression by 36.6% (*p* < 0.01), 79.6% (*p* < 0.0001), and 30.9% (*p* < 0.1), respectively. Simultaneously, NF-LCE enhanced antioxidant defence pathways by modulating the expression of GPX4 and Nrf2 by 0.8-fold increase (*p* = 0.0301) and about 0.8-fold increase (*p* = 0.0385).

## 4. Discussion

Skin ageing is one of the most obvious signs of ageing and is controlled by several endogenous and external variables. UV radiation is the main environmental factor that accelerates skin ageing, termed skin photoaging. The mechanism of UV-induced photoaging is complex and not yet fully understood; however, it is intimately connected to skin cell damage caused by oxidative stress and inflammation [49]. Ferroptosis arises from dysregulated iron homeostasis, where elevated Fe^2+^ levels generate reactive hydroxyl radicals via the Fenton and Haber–Weiss reactions [51]. Based on existing literature [33,34,35,36], this study aimed to induce ferroptosis-related inflammation in HaCaT cells by overloading them with varying incremental iron concentrations. Figure 4 illustrates a linear correlation between an increasing concentration of iron and the levels of LI but a decrease in ferritin storage at 100 µM iron concentration. This observation is consistent with findings from previous studies, indicating a decline in ferritin storage caused by increasing iron concentrations. This phenomenon occurs when the synthesis and storage capacity of ferritin is exceeded, resulting in an increase in free iron within the LI pool [52]. Moreover, during oxidative stress, iron may be released from ferritin either through reductive processes or due to the proteolytic degradation of ferritin [35,52]. Based on observations from optimisation experiments (summarised in Figure 4), a concentration of 100 µM was selected for further investigations, as it provided the optimal balance between inducing iron overload and preserving cell viability. The aim was to stimulate iron-related inflammation without adversely affecting cell viability, as shown in Figure 5. Although the MTT assay provided initial insights into the effect of iron overload on cell viability, we recognise that this parameter alone is insufficient to confirm ferroptosis induction. Future studies will include the assessment of specific ferroptosis markers such as lipid peroxidation (e.g., C11-BODIPY staining), ferrous iron levels (Fe^2+^), and LDH release or PI staining to evaluate membrane integrity. Moreover, the use of ferroptosis inhibitors like ferrostatin-1 or liproxstatin-1 will be instrumental in validating the ferroptotic nature of cell death under iron overload conditions. Finally, while it was sufficient for the purposes of our study by providing indicative trends, equating ferritin protein levels directly with iron content (Figure 4c) may be a simplification. In future studies focusing more on iron uptake and cycling dynamics, we will aim to employ more precise techniques (e.g., mass spectrometry or Mössbauer spectroscopy) to better characterize intracellular iron speciation.

There is a growing trend toward using natural compounds in the realm of skin protection. Despite the promising biological activities of these compounds, their wider application is hindered by factors such as volatility, instability, and insolubility. Nanoformulation has the potential to enhance the stability of these compounds by minimizing volatility and shielding them from environmental influences like oxygen, light, moisture, and pH fluctuations [53,54,55].

In a recent study, Gubitosa et al., 2024 [29] revealed triterpenic acids as primary compounds in a callus culture derived from the pulp of the Annurca Campana apple. The hydroalcoholic extract from this culture (LCE) exhibited notable antioxidant and anti-inflammatory properties [29]. In this work, we aimed to nanoformulate both the LCE and the pure standard, TA, to assess their effects using the ferroptosis model that we optimised.

Prior to nanoencapsulation of LCE and TA, an MTT cell viability assay was performed on HaCaT cells to determine the optimal extract concentrations for subsequent experiments. Based on the MTT assay results (Figure 2), the concentrations of 3.25 μM, 1.625 μM, and 0.8125 μM were selected to be encapsulated. In fact, at these concentrations, an increase in the cell viability was observed after 48 h (Figure 2). The observed increase in HaCaT cell viability following LCE treatment may be attributed to the presence of pentacyclic triterpenes, particularly tormentic acid, within the extract. These compounds have been shown to activate pro-survival signalling pathways, including Akt/ERK and Nrf2, which enhance cell proliferation and protect against oxidative stress-induced cytotoxicity [13,14,15,16,21,56]. In particular, tormentic acid has been reported to promote dermal cell proliferation and exert anti-inflammatory and cytoprotective effects [57].

For the nanocarrier delivery systems of LCE and TA, the polymers Pluronic^®^ F127 and Soluplus^®^ were utilised in a modified thin-film hydration process. Both Soluplus^®^ and Pluronic^®^ F127 are notable as drug solubilisers and absorption and permeation enhancers in formulations such as solid dispersions, micelles, and liposomes. Soluplus^®^ is an amphiphilic non-ionic surfactant synthesised from a polyvinyl caprolactam-polyvinyl acetate-polyethylene glycol graft copolymer that rapidly forms nanocolloidal micelles in aqueous environments, lowering free energy above the critical micellar concentration (CMC). This characteristic makes it a promising carrier for nanodelivery systems, with enhanced bioavailability and improved dissolution profiles. Recent studies highlight its effectiveness in improving poorly water-soluble drugs whilst enabling sustained release [58,59]. In contrast, Pluronic^®^ F127, composed of PEG97-PPO69-PEG97, is a versatile triblock copolymer that is commonly used as a nanocarrier material due to its safety profile, solubilizing capacity, and ability to prolong circulation time, and its pharmaceutical and medical applications were approved by the FDA [60]. Additionally, Pluronic^®^ F127-based formulations have been shown to enhance the topical bioavailability of encapsulated compounds [61,62,63].

Preliminary evaluations were conducted to assess the biological activity of the nanoformulated compound. An MTT cell viability assay was initially performed to determine whether the nanocarrier delivery systems impacted cell survival. The findings indicate that neither the pure compounds nor the encapsulated formulations significantly affected the viability of HaCaT cells (Figure 3). This confirms that both components are biocompatible, non-toxic, and well-tolerated by the skin [64,65]. Based on preliminary assessments (unpublished data), a TA concentration of 3.25 µM for LCE, TA, NF-LCE, and NF-TA was selected for nanoencapsulation for further experiments. The aim was to assess whether the nanoformulated compounds (NF-LCE and NF-TA) and their unformulated counterparts (LCE and TA) could mediate iron-induced inflammation by examining gene expression. Ferroptosis exhibits unique genetic characteristics distinct from other established forms of regulated cell death. The main function of prostaglandin-endoperoxide synthase 2 (PTGS2/COX2) is metabolising arachidonic acid into prostaglandins. This gene is upregulated during ferroptosis, making cells more susceptible to ferropototic attributes. On the other hand, the GPX4 gene is downregulated during ferroptosis. GPX4 converts cytotoxic lipid peroxides into their corresponding alcohols in the presence of glutathione. When GPX4 activity is suppressed, lipid peroxides accumulate, leading to ferroptosis [6,7]. The transcription factor Nrf2 is vital for maintaining skin health and addressing various skin disorders. Present in all skin cell types, Nrf2 regulates oxidative stress, protecting cells from damage. Its short-term activation is crucial for preventing apoptosis caused by UVA and UVB rays. Nrf2 also controls anti-apoptotic molecules that respond to oxidative stress, particularly in the outer layers of the skin exposed to UV sunlight [66]. Nrf2 is also implicated in neutralizing ferroptosis by the transactivation of downstream target genes, mainly in four aspects: iron metabolism, intermediate metabolism, glutathione metabolism, and anti-oxidative stress response [67]. The data presented in Figure 6 demonstrates that iron significantly boosts the expression levels of COX2 while concurrently decreasing the levels of GPX4 and Nrf2. This suggests that iron may play a pivotal role in regulating these proteins, potentially influencing detrimental oxidative stress responses. In contrast, NF-LCE formulations exhibit a remarkable ability to counteract this effect by effectively reversing the increased COX2 expression and restoring the levels of GPX4 and Nrf2. This indicates that NF-LCE could have therapeutic potential in mitigating the impacts of iron overload on cellular processes. Elevated concentrations of intracellular iron can lead to an increase in the expression and activity of inflammatory cytokines such as IL-6 and TNF-α [68,69]. This suggests that when iron levels within cells are high, there is a corresponding rise in the activity of these pro-inflammatory cytokines, which play a crucial role in the body’s inflammatory response. In contrast, NF-LCE has been shown to significantly reduce the expression of IL-6 and TNF-α, indicating that it may have a protective effect against inflammation driven by excess iron. The novelty of this study thus lies in the development of an optimised nanocarrier delivery system to maximise the efficacy of LCE to counteract ferroptosis-mediated inflammation in skin keratinocytes.

## 5. Conclusions

Nanoformulations incorporating pure LCE and standard TA were developed for the first time using Soluplus^®^ and Pluronic^®^ F127, demonstrating favourable physicochemical and cell viability characteristics. A cellular model was optimised to assess the potential of these formulations to mitigate the oxidative and inflammatory stress induced by iron overload. Results revealed that NF-LCE exerted a protective effect by reducing the expression of inflammatory genes and supporting antioxidant defence mechanisms. However, due to limitations in the current experimental design, particularly in the characterisation of ferroptosis, these findings should be considered preliminary. Further investigations using specific ferroptosis markers and validated inhibitors are required to confirm whether the observed effects involve modulation of ferroptotic pathways. Altogether, our study supports the potential of LCE as a bioactive phytochemical and highlights the added value of nanoformulation in enhancing its therapeutic profile. Future studies in advanced in vitro models and eventual clinical translation will be necessary to fully explore its role in skin health as a topical nutraceutical.

## Figures and Tables

**Table 1:** Nucleotide sequences of the primers used in real-time PCR.

	Sequence	Accession Number
GPX4	F-5′-ACAAGAACGGCTGCGTGGTGAA-3′ R-5′-GCCACACACTTGTGGAGCTAGA-3′	NM_001367832.1
COX2	F-5′-CGGTGAAACTCTGGCTAGACAG-3′ R-5′-GCAAACCGTAGATGCTCAGGGA-3′	NM_000963.4
NrF2	F-5′-AAACCAGTGGATCTGCCAAC-3′ R-5′-ACGTAGCCGAAGAAACCTCA-3′	NM_001313902.2
IL-6	F-5′-GGTACATCCTCGACGGCATCT-3′ R-5′-GTGCCTCTTTGCTGCTTTCAC-3′	NM_000600.5
TNF-α	F-5′-CTCTTCTGCCTGCTGCACTTTG-3′ R-5′-ATGGGCTACAGGCTTGTCACTC-3′	NM_000594.4
36B4	F-5′-CGACCTGGAAGTCCAACТAC-3′ R-5′-ATCTGCTGCATCTGCTTG-3′	NM_053275.4

**Table 2:** Mean particle size, PDI, and zeta potential of the formulations. Data represent the mean ± SD of three replicates.

Formulation Name	Particle Size (nm)	PDI	Zeta Potential (mV)
NF-Blank	75.00 ± 0.47	0.02 ± 0.01	−5.24 ± 0.69
NF-TA	81.48 ± 0.39	0.06 ± 0.01	−4.01 ± 0.36
NF-LCE	103.17 ± 0.87	0.21 ± 0.00	−1.88 ± 0.64

## Data Availability

The data supporting this study’s findings are available from the corresponding author, M.G.Z., upon reasonable request.

## Supplementary Materials

The following supporting information can be downloaded at: https://www.mdpi.com/article/10.3390/antiox14060631/s1, Table S1. Three thermal stage cycles utilised in real-time PCR.
