# Supplementary material for: Evaluation of Antioxidant and Anti-Inflammatory Effects of a Nanoformulation Derived from Annurca Apple Callus Extract in an In Vitro Model of Iron Overload-Induced Inflammation
Source: Antioxidants (Basel). 2025 May 24;14(6):631. doi: 10.3390/antiox14060631 (PMC12189543; doi:10.3390/antiox14060631)
Supplement: Supplementary file 1 [file antioxidants-14-00631-s001.zip › antioxidants-3597140-supplementary.pdf]

Supplementary Table S1. Three thermal stage cycles utilised in real-time PCR

**Stage 1**	Initial Denaturation	Rep: 1	95°C	30sec
**Stage 2**	Cycling Reaction	Reps: 40	95°C<br>60°C	3-10sec<br>10-30sec
**Stage 3**	Melting Curve	Rep: 1	95°C<br>60°C<br>95°C	15sec<br>60sec<br>15sec
